# Histopathological modeling of status epilepticus-induced brain damage based on *in vivo* diffusion tensor imaging in rats

**DOI:** 10.3389/fnins.2022.944432

**Published:** 2022-07-29

**Authors:** Isabel San Martín Molina, Raimo A. Salo, Olli Gröhn, Jussi Tohka, Alejandra Sierra

**Affiliations:** A.I. Virtanen Institute for Molecular Sciences, University of Eastern Finland, Kuopio, Finland

**Keywords:** cell counting, diffusion tensor imaging, predictive modeling, structure tensor analysis, astrocyte morphology

## Abstract

Non-invasive magnetic resonance imaging (MRI) methods have proved useful in the diagnosis and prognosis of neurodegenerative diseases. However, the interpretation of imaging outcomes in terms of tissue pathology is still challenging. This study goes beyond the current interpretation of *in vivo* diffusion tensor imaging (DTI) by constructing multivariate models of quantitative tissue microstructure in status epilepticus (SE)-induced brain damage. We performed *in vivo* DTI and histology in rats at 79 days after SE and control animals. The analyses focused on the corpus callosum, hippocampal subfield CA3b, and layers V and VI of the parietal cortex. Comparison between control and SE rats indicated that a combination of microstructural tissue changes occurring after SE, such as cellularity, organization of myelinated axons, and/or morphology of astrocytes, affect DTI parameters. Subsequently, we constructed a multivariate regression model for explaining and predicting histological parameters based on DTI. The model revealed that DTI predicted well the organization of myelinated axons (cross-validated R = 0.876) and astrocyte processes (cross-validated R = 0.909) and possessed a predictive value for cell density (CD) (cross-validated R = 0.489). However, the morphology of astrocytes (cross-validated R > 0.05) was not well predicted. The inclusion of parameters from CA3b was necessary for modeling histopathology. Moreover, the multivariate DTI model explained better histological parameters than any univariate model. In conclusion, we demonstrate that combining several analytical and statistical tools can help interpret imaging outcomes to microstructural tissue changes, opening new avenues to improve the non-invasive diagnosis and prognosis of brain tissue damage.

## Introduction

In recent years, diffusion magnetic resonance imaging (dMRI) methods, such as diffusion tensor imaging (DTI), have become widely used tools to study the brain’s response to pathological insults in both clinical and research settings (Alexander et al., 2007; Tae et al., 2018; Müller et al., 2020). The sensitivity of dMRI is based on the diffusion of water molecules, which reflects the tissue microenvironment of the cellular components, such as cell bodies, neurites, axons, or blood vessels. Neurodegeneration, axonal plasticity and injury, inflammation, or dendritic remodeling are some factors that can potentially alter the tissue microenvironment and be detected by DTI parameters (Laitinen et al., 2010; Haber et al., 2017; Robinson et al., 2017; Benjamini et al., 2020; Clément et al., 2020; Graham et al., 2020; Tavor et al., 2020). However, the complexity of these cellular alterations complicates the interpretation of DTI parameters, at present, their characterization is far from satisfactory.

Changes in DTI parameters in the diseased brain have been assessed with histology. Histological stainings and quantitative analyses are known to provide morphological information on the cellular components and their changes occurring during pathological conditions (Herrera et al., 2008; Augustinack et al., 2010; Concha et al., 2010; Flint et al., 2010; Chang et al., 2017). Traditional quantitative analyses, such as optical density, counting, or stereological-based methods, have been used to extract tissue-derived parameters that can be correlated to dMRI parameters (Trivedi et al., 2009; Jespersen et al., 2010; Laitinen et al., 2010; Bennett et al., 2012; Janz et al., 2017; Göbel-Guéniot et al., 2020). Recently, advanced histological analysis methods have introduced new ways to quantify tissue parameters directly comparable to dMRI parameters, e.g., Fourier transform- and structure tensor (ST)-based analyses that extract anisotropy, orientation, or dispersion data (Budde et al., 2011; Budde and Frank, 2012; Salo et al., 2017, 2021; Breu et al., 2019; San Martín Molina et al., 2020). Moreover, specific histological methods for quantifying the morphology of glial cells in both the resting and activated states (Avignone et al., 2015; Lanjakornsiripan et al., 2018; Young and Morrison, 2018; Clément et al., 2020), can provide new insights into the relationship between dMRI parameters and changes occurring in the tissue during pathological conditions. However, it remains a major challenge in the field to determine how DTI parameters could be associated with one and/or several tissue changes, and more importantly, how imaging data would be able to predict the tissue changes in response to pathological conditions.

This present study describes how the interpretation of DTI can be improved by utilizing a multivariate statistical model of tissue microstructure and assessing the relationship between several DTI and histological parameters. The model evaluates if *in vivo* DTI can explain and predict the underlying microstructural tissue changes after status epilepticus (SE) induced-brain damage. *In vivo* DTI was performed 79 days after SE, subsequently, the brains were prepared for histology. Previous studies have described brain histopathological changes after SE in rats, such as axonal sprouting (Kuo et al., 2008; Laitinen et al., 2010; Sierra et al., 2011), axonal and astrocytes reorganization (Salo et al., 2017), neurodegeneration and gliosis (Sierra et al., 2015), or white matter alterations (Sierra et al., 2011; van Eijsden et al., 2011; Luna-Munguia et al., 2021). Here, we evaluated the cytoarchitecture and axonal and astrocyte morphology in controls and SE rats using automated cell counting, ST-, and morphological skeleton-based analyses in the corpus callosum, layers V and VI of the parietal cortices, and hippocampal subfield CA3b. As a novelty in our study, we performed estimation-based statistics to assess the effects of SE in both DTI and histological parameters. Moreover, we constructed a regression model based on DTI, which could predict tissue microstructural alterations after SE, by adopting a leave-one-animal out and leave-one-brain region out cross-validation (CV). Thus, this study combines several brain areas and tissue morphological parameters together with advanced analytical and statistical tools, which improve the interpretation of DTI outcomes in the damaged brain.

## Materials and methods

### Animals and status epilepticus model

Adult male Wistar rats were used in all the experiments (10 weeks old, 300–350 g, National Laboratory Animal Center, Kuopio, Finland), housed individually in cages in a climate-controlled room with an *ad libitum* diet. All animal procedures were approved by the Animal Ethics Committee of the Provincial Government of Southern Finland and performed in accordance with the guidelines set by the European Community Council Directives 2010/63/EEC.

All the experimental procedures and data acquisition were as described in Salo et al. (2017). Briefly, we induced SE, which models temporal lobe epilepsy, by injection of kainic acid (i.p., 10 mg/kg, K2389, Sigma-Aldrich, St. Louis, MO, United States; *n* = 14) or pilocarpine (s.c., 1 mg/kg, #S-8502, Sigma-Aldrich; *n* = 14). The control group was treated with an injection of 0.9% NaCl (*n* = 4). The development of SE and its severity score were assessed within 3 h after the injections (Racine, 1972). After kainic acid injection, seven animals died during or after SE, and one animal did not exhibit signs of SE (not included in the study). In the pilocarpine group, diazepam was administered 120 min after the appearance of SE to reduce mortality. After pilocarpine injection, six animals died, and one animal did not show signs of SE (not included in the study). Altogether, the total number of surviving SE animals included in this study, and that had experienced recurrent generalized seizures for at least 30 min, was six in the kainic acid group and seven in the pilocarpine group.

### *In vivo* diffusion tensor imaging acquisition and data processing

During the MRI scans, the animals were under 1.0–1.5% isoflurane anesthesia, breathing 70% nitrogen/30% oxygen, and had a stable body temperature (approximately 37°C). *In vivo* DTI was conducted using a horizontal 7-T Bruker PharmaScan MRI system (Bruker BioSpin, Germany) with an actively decoupled quadrature volume transmitter coil and a quadrature rat receiver surface coil. In the acquisition of *in vivo* DTI data, we used a diffusion-weighted segmented spin-echo planar imaging pulse sequence with the following parameters: TE = 30 ms, TR = 2.5 s, number of averages = 32, number of segments = 4, 21 directions (Δ = 11 ms, δ = 4 ms, *b*-value = 1,000 s/mm^2^), FOV of 21.2 × 14.08 mm^2^ (covered with 192 × 128 points, resolution of 110 × 110 μm^2^), number of slices = 14, slice thickness = 500 μm, and scan time = 2 h 20 min. These animals were part of a longitudinal study (Salo et al., 2017), and in the present study, we used data from the last time point, 79 days, which corresponded to histology.

In the DTI data processing, all data were corrected for motion and eddy current distortions with FMRIB’s Linear Image Registration Tool (FLIRT) (Jenkinson and Smith, 2001; Jenkinson et al., 2002) in the FMRIB Software Library (FSL 4.0).^1^ After eddy current corrections, the diffusion tensors, and their respective eigenvalues (λ_1_, λ_2_, and λ_3_) were determined using FSL. Then, we generated the fractional anisotropy (FA), axial diffusivity (AD), radial diffusivity (RD), and mean diffusivity (MD) maps (Basser and Pierpaoli, 1996; Pajevic and Pierpaoli, 1999). We also calculated linear (CL), planar (CP), and spherical (CS) anisotropy indices (Westin et al., 2002), which provide additional information related to changes in the anisotropic diffusion geometry in the tissue microstructure, as previously demonstrated post-SE (Salo et al., 2017).

### Tissue processing for histology and stainings

After the scans, all the rats were deeply anesthetized by an i.p. injection (6 ml/kg) with an anesthetic cocktail containing sodium pentobarbital (58 mg/kg), chloral hydrate (60 mg/kg), magnesium sulfate (127.2 mg/kg), propylene glycol (42.8%), and absolute ethanol (11.6%). The deep level of anesthesia was kept by 5% isoflurane (70/30 N_2_/O_2_) followed by transcardial perfusion, first with 0.9% NaCl, and then, by 4% paraformaldehyde (PFA). The brains were removed from the skull and postfixed in 4% PFA for 4 h. Then, the brains were placed in a cryoprotective solution [20% glycerol in 0.02 M potassium phosphate-buffer saline (KPBS), pH 7.4 for 36 h]. The brains were frozen in dry ice and stored at −70°C until cutting. The brains were sectioned in a sliding microtome (coronal plane, 30 μm, 1-in-5 series). We stored the first series of sections in 10% formalin, and the remaining series in cryoprotectant-tissue collecting solution (30% ethylene glycerol, 25% glycerol in 0.05 M sodium phosphate buffer) at −20°C until processing.

The first series of sections were stained with Nissl (thionin) to assess the cytoarchitectonics and severity of tissue damage after SE. The second series of sections were stained for myelin using gold chloride to examine the myeloarchitecture (Laitinen et al., 2010). Briefly, we incubated the sections mounted on gelatin-coated slides in a 0.2% gold chloride solution (HAuCl_4_⋅3H_2_O, G-4022 Sigma-Aldrich, Finland) in 0.02 M sodium phosphate buffer (pH 7.4) containing 0.09% NaCl for 3–4 h in dark at room temperature (RT). After washing in 0.02 M sodium phosphate buffer containing 0.09% NaCl, the sections were incubated in 2.5% sodium thiosulfate solution (5 min). Then, the sections were washed in the buffer solution, dehydrated in an ascending ethanol series, cleared in xylene, and cover-slipped with DePeX (BDH, Laboratory Supplies, Dorset, United Kingdom).

From the third series of sections, we stained three consecutive sections immunohistochemically with an astrocyte marker, glial fibrillary acidic marker (GFAP), to assess the morphology of the astrocytes (Salo et al., 2017). Briefly, free-floating sections were washed in 0.02 M KPBS and incubated in 1% H_2_O_2_ (15 min) to remove endogenous peroxidase activity. After washing in buffer, non-specific binding in the sections was blocked by placing them in 10% normal horse serum (NHS) solution (0.4% Triton X-100, 0.02 M KPBS) for 2 h. Then, the sections were incubated for 48 h (4°C) in the primary antibody (mouse anti-GFAP, 1:4,000, #814369; Boehringer Mannheim, Germany) diluted in 1% NHS, 0.4% Triton X-100 in KPBS. Next, sections were washed and incubated for 2 h at RT in the secondary antibody (biotinylated horse anti-mouse immunoglobulin G, 1:200, BA-2000; Vector Laboratories, Burlingame, CA, United States) solution (1% NHS, 0.4% Triton X-100, KPBS). After washing, the sections were incubated for 1 h at RT in 1% avidin-biotin in KPBS (PK-4000, Vector Laboratories). Then, the sections were placed black into the secondary antibody solution (45 min), followed by immersion in the avidin-biotin solution (30 min). To visualize the secondary antibody, we used a solution containing 0.05% 3′,3′ -diaminobenzidine (#34001, Pierce Chemical, Rockford, IL, United States) and 0.04% H_2_O_2_ in KPBS. After washing in 0.1 M PB, we mounted the sections on gelatin-coated slides and dried them overnight (37°C). The sections were intensified with osmium (OsO_4_, #19170, Electron Microscopy Sciences, Hatfield, PA, United States) and thiocarbohydrazide (#21900, Electron Microscopy Sciences). Finally, the sections were washed with the buffer, dehydrated in an ascending ethanol series, cleared in xylene, and cover-slipped with DePeX.

### Histological data and analyses

We acquired high-resolution photomicrographs of the whole brain in three consecutive Nissl-, myelin-, and GFAP- stained sections with a ZeissAxioImager2 light microscope (White Plains, NY, United States) equipped with a digital camera (Zeiss Axiocam color 506). The images were acquired as tiles with a resolution of 0.013 μm^2^/pixel.

In Nissl-stained sections, we quantified cell density (CD) using an automated cell counting in-house MATLAB code as described in San Martín Molina et al. (2020) and available at.^2^ An increase in CD on Nissl-stained sections was indicative of gliosis as reported in previous studies (Sierra et al., 2015; San Martín Molina et al., 2020). To assess the performance of our automated cell counting analysis, we adjusted the image threshold of 10 randomly selected photomicrographs and compared the results with manual counting performed by an expert (I.S.M.M.). The mean percentage error from the automated cell counting analysis was 11.30% (Supplementary Figure 1). In myelin- and GFAP-stained sections, we used ST-based analysis to calculate the anisotropy index (AI) as a histological derived parameter using the eigenvalues, which were obtained by applying the pixel-wise ST-based method in each image following the same procedure and parameters as previously described in San Martín Molina et al. (2020). Briefly, we first convolved the images with a directional derivative of a 2D Gaussian function in two directions (size = 11 pixels, σ = 3 pixels), to extract the directional derivatives of an image. Then, we formed a tensor from the partial derivatives and summed the STs into a pixelwise ST. Additionally, in GFAP-stained sections, we used a skeleton-based approach to extract morphological parameters of the astrocyte processes by adopting the Analyze Skeleton 2D/3D plugin from the Fiji project in ImageJ software^3^ (ImageJ version 1.53c, National Institutes of Health, United States) as developed by Arganda-Carreras et al. (2010). We optimized the protocol steps to our photomicrographs based on the workflow introduced by Young and Morrison (2018) (Supplementary Figure 2) by visualizing the skeleton-derived morphology in random photomicrographs to ensure that the skeleton plugin worked in our images. Briefly, we removed the noise by first applying a bandpass filter (large structure filter = 40, small structure filter = 3) and removing the background of the images (rolling ball radius = 200 pixels). Then, we adjusted the brightness and contrast (min = 10, max = 254) and used an unsharp mask filter (σ = 1.5 pixels, mask weight = 0.6) to enhance the contrast of the features in the images. Next, we used a despeckle function to remove the noise created from the unsharp mask filter step. Following these steps, we converted the grayscale image to a binary image by thresholding with values between 0 and 223 (corpus callosum = 0–218, layer V of parietal cortex = 0–223, layer VI of parietal cortex = 0–219, CA3b = 0–223). Additionally, we applied three function steps to remove noise and gaps between processes from the binary images (despeckle, close, and remove outliers). Finally, we skeletonized the images using the skeletonize step and ran the AnalyzeSkeleton 2D/3D plugin. From the skeletonized images, we first discarded small fragments remaining after image thresholding, where we determined a minimum cut-off value measuring several random small fragments in the images (cut-off value = 0.7 μm length). Then, we sorted the data according to endpoint voxels from largest to smallest, and by branch length. Subsequently, we removed the values that contained 2 endpoints with a branch length less than the determined cut-off value. Finally, we extracted the following parameters: number of branches defined by slab segments connecting endpoints and junctions’ parameters, branch length, triple points (junctions with three branches), and quadruple points (junctions with four branches). In each skeleton-derived parameter, we summed all the values extracted to obtain the total number. Additionally, we calculated the average length of the astrocyte processes per animal by dividing the total branch length per total number of branches in each image, to assess the overall change in length of the astrocyte processes in each animal.

### Region-of-interest based approach for diffusion tensor imaging and histology

**FIGURE 1:**
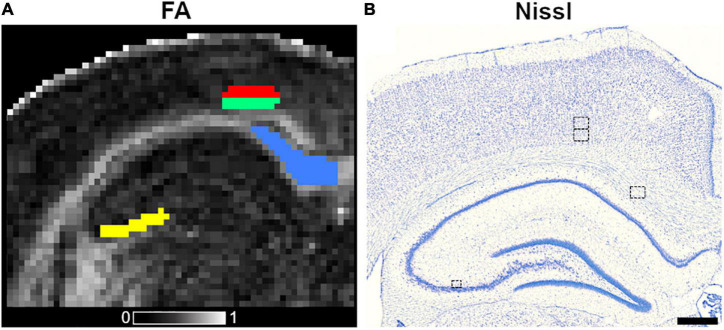
Outlined ROIs in coronal fractional anisotropy (FA) map **(A)** and Nissl-stained section **(B)** from a control animal. The ROIs included in this study are corpus callosum (blue color), subfield CA3b of the hippocampus (yellow), layers V (red), and VI (green) of the parietal cortex, and the equivalent in the Nissl-stained section. The ROI area outlined on the histological photomicrographs in the left corpus callosum, layers V and VI of the parietal cortices was 180.81 × 141.31 μm^2^, whereas the ROI area in CA3b was 104.42 μm^2^ × 89.89 μm^2^. The gray scale reveals FA values between 0 (black) and 1 (white). Scale bar: 500 μm.

The selection of brain areas was based on previous studies. The subfield CA3b of the hippocampus and the corpus callosum have been reported as damaged after SE in rats (Sierra et al., 2011; Salo et al., 2017; Luna-Munguia et al., 2021). The parietal cortex has not been described as a component of the network in the SE model; however, we observed activated astrocytes in GFAP staining on layers V and VI (refer to Results), which motivated the inclusion of these layers in the analyses. We extracted DTI and histological parameters by adopting an region-of-interest (ROI)-based approach. An expert (I.S.M.M.) manually outlined the brain regions mentioned above at −3.60 mm from bregma on the left hemisphere in both DTI maps and histological photomicrographs (Figure 1). We ensured that DTI and histology ROIs were drawn in the same location by utilizing whole brain photomicrographs and anatomical landmarks, such as whole brain, ventricles, shape, and size of white and gray matter, nuclei and layers, to find the corresponding location as in DTI maps. The ROIs outlined in histological sections were representative of the DTI ones. We outlined the ROIs in DTI maps using an in-house Matlab tool called AEDES^4^ (Matlab R2018b; MathWorks, Natick, Massachusetts, United States) (Figure 1A). Similarly as in DTI, we outlined all the ROIs on photomicrographs to extract histological-derived parameters using the ZEN software (version 3.1; Carl Zeiss Microscopy GmbH, Germany) (Figure 1B).

### Statistical analyses

#### Estimation statistics-based approach

In the statistical analyses, we aimed to assess the effect of SE in DTI and histological parameters, reporting the effect sizes of SE-induced brain damage in addition to null hypothesis significance testing and utilizing an estimation statistics-based approach. In the present study, we consider both kainic acid- and pilocarpine-treated animals as one SE animal group for statistical analyses, since both animal models exhibited similar histopathological and DTI profiles based on previous studies (Covolan and Mello, 2000; Salo et al., 2017). We performed estimation statistics using the DABEST package (version 0.3.1) developed by Ho et al. (2019) in Python (version 3.8). As outputs, we quantified the effect sizes with Cohen’s d along with its confidence intervals (CI). The CIs were computed bias-corrected and accelerated bootstrap. In order to simplify the interpretation of the effect sizes, we followed the standard practice to consider Cohen’s |d| = 0.8, |d| = 0.5 and |d| = 0.2 as large, medium, and small effect size, respectively (Cohen, 1988). We complemented the effect size statistics with hypothesis tests using a studentized two-sided permutation *t*-test (100,000 permutations). We applied the multiple comparison correction by using the Benjamini-Hochberg false discovery rate (BH-FDR; Benjamini and Hochberg, 1995) to the *p*-values, but the reported CIs are uncorrected. The plots for both MRI and histological parameters represent the values expressed as mean with 95% CI, using GraphPad Prism (version 5.03, GraphPad Software Inc., La Jolla, CA, United States).

#### Multiple linear regression and Pearson correlation analyses

When evaluating the relationship between DTI and histology in the selected brain regions, we performed a multiple linear regression in an attempt to explain histological parameters based on DTI parameters. The model that we used is

$$y_{k\,j}={\text{b}}^{T}\,{\text{x}}_{k\,j}+c+e_{k\,j}$$

where *y*_*kj*_ is a histological parameter for animal *k* in the region *j* (corpus callosum, layer V, layer VI, CA3b), ***x***_*kj*_ is the vector of DTI parameters (FA, RD, MD, CP, CS) for animal *k* in the region *j*, ***b*** and *c* are the regression parameters, and *e*_*kj*_ represents normally distributed independent and identically distributed errors. We concatenated parameters from all the regions and all the animals into a single model.

We excluded AD and CL from the DTI parameters to avoid a singular variance-covariance matrix as AD = 3*MD - 2*RD and CL = 1 – CP – CS. We further noted that all DTI parameters were non-linear functions of just three eigenvalues of the diffusion tensor. Therefore, the five remaining DTI parameters displayed a notable amount of structural collinearity, which means that the inferences concerning individual coefficients of the regression model were not reliable. However, the five-parameter model had a non-singular variance-covariance matrix, and the explanatory and predictive accuracy of the model could be evaluated. The choice of AD and CL as the parameters to be removed was arbitrary and had no influence on the evaluation of the model’s accuracy. We used SPSS to fit the regression model and estimated the 95% CI values for *R*^2^ using the SPSS code from Smithson (2001). Due to the structural collinearity of DTI parameters, we assessed the relationships between individual DTI (FA, RD, MD, CP, and CS) and histological parameters in all selected brain regions using Pearson correlation analysis with GraphPad Prism (version 5.03, GraphPad Software Inc., La Jolla, CA, United States). We report the correlation coefficient R along with 95% CI and applied multiple comparison corrections based on the *p*-values (i.e., CIs are uncorrected). Moreover, we also report the F statistics to support that the regression model provides a better fit than a model without independent variables. The 95% CI values were computed as accelerated bootstraps in SPSS (version 27, IBM SPSS Statistics, United States).

#### Cross-validation by leaving-one-animal out and by leaving-one-brain region out

We assessed how predictive DTI would be with regard to histology by evaluating the performance of the regression model by running leave-one-animal out and leaving-one-region out CV. Since the regression model (incorrectly) assumes that the parameters from different regions of the same animal are independent, we evaluated its predictive performance using leave-one-animal out CV. We report the quality of validated predictions using a cross-validated correlation coefficient R and the coefficient of determination (*Q*^2^) of the relationship between the two has been analyzed in detail in Moradi et al. (2017). *Q*^2^ is defined by $Q^{2}=1-\frac{\left(1/K\,J\right)\,\sum_{k}\sum_{j}{\left(y_{k\,j}-\hat{y_{k\,j}}\right)}^{2}}{\left(1/K\,J\right)\,\sum_{k}\sum_{j}{\left(y_{k\,j}-\bar{y_{k\,j}}\right)}^{2}}$, where *y*_*kj*_ is the true histological parameter, $\hat{y_{k\,j}}$ is the predicted histological parameter of the animal *k* in the region *j* and $\bar{y_{k\,j}}$ is the average of the true histological parameter values. CV-analyses were performed by using an in-house MATLAB code available at.^5^

## Results

### Effects of status epilepticus-induced brain damage in diffusion tensor imaging and histological parameters

**FIGURE 2:**
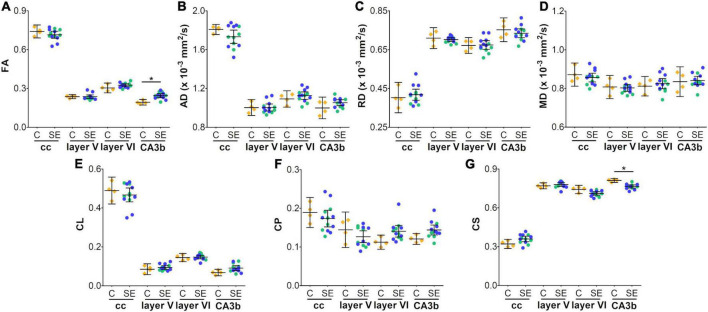
*In vivo* DTI parameters in control and status epilepticus animals at 79 days **(A–G)**. Controls are represented as yellow diamonds, kainic acid-treated as green, and pilocarpine-treated as blue circles, respectively. The bars represent mean values with 95% CI. Differences between C and SE animals are denoted with asterisks (BH-FDR-corrected *q*-value * < 0.05; two-sided permutation *t*-test). SE animals exhibited an increase in FA **(A)** or a decrease in CS **(G)** parameters in the subfield CA3b as compared to controls. AD, axial diffusivity; C, control; CA, cornus ammonis; cc, corpus callosum; CL, linear anisotropy; CP, planar anisotropy; CS, spherical anisotropy; FA, fractional anisotropy; MD, mean diffusivity; RD, radial diffusivity; SE, status epilepticus.

**FIGURE 3:**
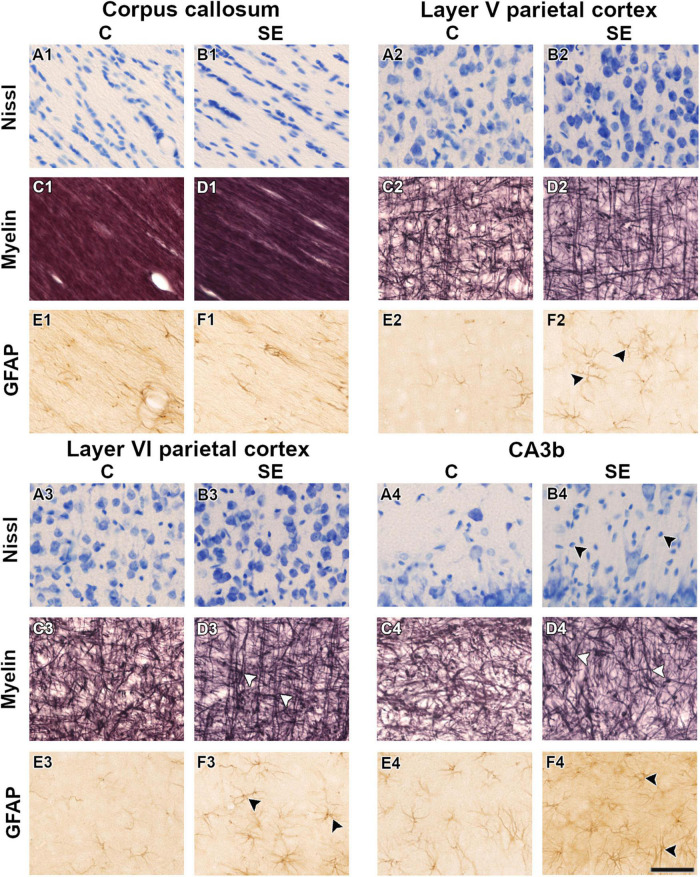
Representative high-magnification photomicrographs in Nissl-, myelin-, and GFAP-stained sections of one control **(A,C,E)** and one status epilepticus **(B,D,F)** animal in white and gray matter areas. White arrowheads indicate changes in the organization of myelinated axons **(D3,D4)**. Black arrowheads indicate increased cellularity **(B4)** and an increase in the number of astrocyte processes and length **(F2,F3,F4)** at 79 days post-SE. The same animals are shown in the three stainings. Scale bar: 50 μm. C, control; CA, cornus ammonis; GFAP, glial fibrillary acidic protein; SE, status epilepticus.

**FIGURE 4:**
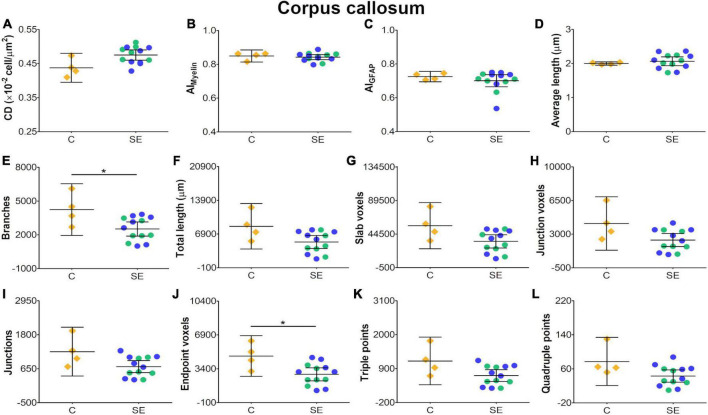
Histological-derived parameters from automated cell counting analyses **(A)**, structure tensor (ST)-based analyses of myelin **(B)**, and ST **(C)** and skeleton-based analysis **(D–L)** from GFAP-stained sections in the corpus callosum. Controls are represented as yellow diamonds, kainic acid-treated as green, and pilocarpine-treated as blue circles, respectively. The bars represent the mean values with 95% CI. Differences between C and SE animals are denoted with asterisks (BH-FDR-corrected *q*-value * < 0.05; two-side permutation *t*-test). SE animals exhibited decreases in branches **(E)** and endpoint voxels **(J)** parameters as compared to controls. AI, anisotropy index; C, control; CD, cell density; SE, status epilepticus.

In the corpus callosum, the effect of SE was large in CS [|d| = 1.160, 95% CI (0.23, 2.32); Table 1 and Figure 2G] and medium in FA and AD (Table 1 and Figures 2A,B). However, CIs for FA and AD were wide, which was evidence of uncertainty about the effect of SE in these DTI parameters (Table 1). Despite no apparent changes in cellularity or the morphology of axons or astrocyte processes between SE and control animals (Figures 3A1–F1), the effect of SE was large in CD [|d| = 1.480, 95% CI (0.01, 2.70); Table 2 and Figure 4A] and in all extracted skeleton-based parameters (|d| > −0.8; Table 2 and Figures 4E–L), except for average length (Table 2 and Figure 4D). It is important to highlight that although the effect size of SE was large in all skeleton-based parameters, wide CIs are indicative of uncertainty regarding the effect size in these histological parameters. In AI_*Myelin*_ and AI_*GFAP*_, the effect of SE was small (Figures 4B,C).

**TABLE 1:** Effect of SE-induced brain damage in quantitative ROI-based analyses of DTI parameters.

	cc	Layer V	Layer VI	CA3b
	
DTI parameters	Cohen’s |d|(95% CI) *q*	Cohen’s |d|(95% CI) *q*	Cohen’s |d|(95% CI) *q*	Cohen’s |d|(95% CI) *q*
FA	−0.596 (−1.45, 0.43) 0.739	0.029 (−0.89, 0.84) 0.978	1.080 (−0.59, 2.42) 0.286	2.09 (0.98, 3.22)* 0.026
AD	−0.726 (−1.37, 0.05) 0.620	0.018 (−1.30, 0.94) 0.978	0.559 (−0.30, 1.87) 0.740	0.993 (−0.26, 2.64) 0.322
RD	0.312 (−1.17, 1.32) 0.763	−0.364 (−2.23, 1.93) 0.746	0.077 (−0.81, 1.14) 0.962	−0.479 (−1.83, 0.62) 0.740
MD	−0.402 (−1.84, 0.54) 0.746	−0.149 (−1.51, 1.23) 0.895	0.318 (−0.50, 1.49) 0.763	0.154 (−1.29, 1.66) 0.895
CL	−0.402 (−1.26, 0.69) 0.746	0.530 (−0.64, 1.77) 0.740	0.165 (−0.95, 1.32) 0.895	1.150 (0.41, 1.92) 0.239
CP	−0.465 (−1.62, 0.45) 0.740	−0.691 (−1.85, 0.74) 0.620	1.250 (0.53, 2.12) 0.239	1.210 (0.40, 1.99) 0.239
CS	1.160 (0.23, 2.32) 0.239	0.422 (−0.50, 1.46) 0.740	−1.430 (−2.48, −0.01) 0.239	−2.560 (−3.71, −1.33)* 0.013

The effect of SE- The effect of SE-induced brain damage was large in CS in the corpus callosum; in FA, CP, and CS in layer VI of the parietal cortex; in FA, AD, CL, CP, and CS in the subfield CA3b. BH-FDR-corrected q-values are denoted with asterisks (*q < 0.05; two-side permutation *t*-test). AD, axial diffusivity; CA, cornus ammonis; cc, corpus callosum; CI, confidence interval; CL, linear anisotropy; CP, planar anisotropy; CS, spherical anisotropy; DTI, diffusion tensor imaging; FA, fractional anisotropy; MD, mean diffusivity; RD, radial diffusivity.

**TABLE 2:** Effect of SE-induced brain damage in quantitative ROI-based analyses of histological parameters.

	cc	Layer V	Layer VI	CA3b
Histological parameters	Cohen’s |d|(95% CI) *q*	Cohen’s |d|(95% CI) *q*	Cohen’s |d|(95% CI) *q*	Cohen’s |d|(95% CI) *q*
AI_*Myelin*_	−0.275 (−1.15, 1.31) 0.690	0.512 (−0.67, 1.91) 0.546	0.201 (−0.54, 1.00) 0.760	2.300 (0.45, 4.34)* 0.012
AI_*GFAP*_	−0.452 (−0.94, 0.51) 0.590	0.273 (−0.75, 1.51) 0.690	1.350 (0.20, 2.70) 0.068	1.650 (0.80, 3.14)* 0.044
CD	1.480 (0.01, 2.70) 0.051	−0.476 (−1.49, 0.58) 0.569	0.120 (−0.60, 1.26) 0.848	1.250 (0.41, 2.71) 0.071
Average length	0.340 (−0.42, 1.03) 0.627	0.421 (−1.86, 2.91) 0.590	2.060 (0.96, 3.57)* 0.018	0.058 (−0.68, 0.93) 0.920
Branches	−1.520 (−2.78, −0.22)* 0.047	2.580 (1.50, 4.01)** 0.001	1.550 (0.37, 2.71)* 0.047	0.503 (−0.69, 1.22) 0.547
Branch length	−1.370 (−2.62, −0.16) 0.068	3.430 (1.87, 5.12)** 0.001	1.800 (0.80, 2.82)* 0.031	0.576 (−0.70, 1.40) 0.490
Slab voxels	−1.340 (−2.59, −0.14) 0.068	3.510 (1.88, 5.31)*** 2.400 × 10^–6^	1.830 (0.83, 2.84)* 0.029	0.603 (−0.67, 1.44) 0.464
Junction voxels	−1.360 (−2.78, −0.17) 0.068	2.390 (1.47, 3.76)** 0.001	1.500 (0.45, 2.46)* 0.047	0.388 (−0.98, 1.05) 0.596
Junctions	−1.330 (−2.73, −0.09) 0.068	2.400 (1.42, 3.67)** 0.003	1.520 (0.49, 2.49)* 0.047	0.394 (−0.96, 1.04) 0.596
Endpoint voxels	−1.630 (−2.84, −0.27)* 0.044	2.620 (1.53, 3.98)** 0.003	1.520 (0.14, 2.94)* 0.047	0.752 (−0.23, 1.61) 0.320
Triple points	−1.330 (−2.74, −0.05) 0.068	2.470 (1.48, 3.75)*** 2.400 × 10^–6^	1.560 (0.54, 2.53)* 0.047	0.396 (−0.94, 1.05) 0.596
Quadruple points	−1.280 (−2.83, −0.32) 0.072	1.790 (0.75, 2.81)* 0.029	0.989 (−0.29, 1.91) 0.156	0.368 (−1.09, 1.02) 0.596

The effect of SE-induced brain damage was large in CD and all skeleton-based parameters in the corpus callosum; in all skeleton-based parameters in layer V of the parietal cortex; AI_*GFAP*_, average length and in all skeleton-based parameters in layer VI of the parietal cortex; in AI_*Myelin*_, AI_*GFAP*_ and CD in the subfield CA3b. BH-FDR-corrected *q*-values are denoted with asterisks (*q < 0.05; **q < 0.01; ***q < 0.001; two-side permutation t-test). AI, anisotropy index; C, control; CA, cornus ammonis; cc, corpus callosum; CD, cell density; CI, confidence interval.

**FIGURE 5:**
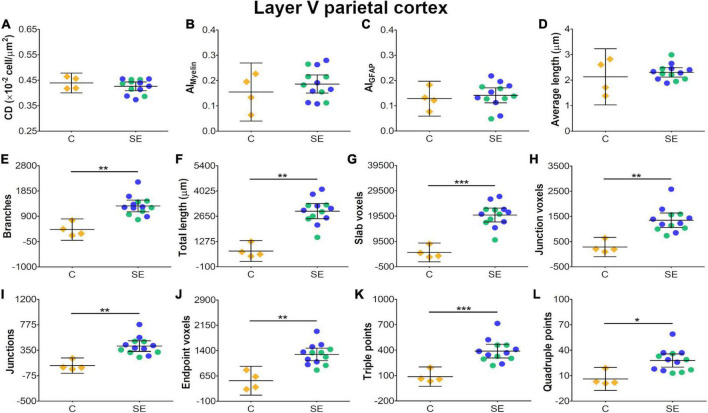
Histological-derived parameters from automated cell counting analyses **(A)**, structure-tensor (ST)-based analyses of myelin **(B)**, and ST **(C)** and skeleton-based analysis **(D–L)** from GFAP-stained sections in layer V of the parietal cortex. Notations as in Figure 4. SE animals revealed increases in all skeleton-based parameters **(E–L)** as compared to controls (BH-FDR-corrected *q*-values * < 0.05, ** < 0.01, *** < 0.001; two-side permutation *t*-test). AI, anisotropy index; C, control; CD, cell density; SE, status epilepticus.

**FIGURE 6:**
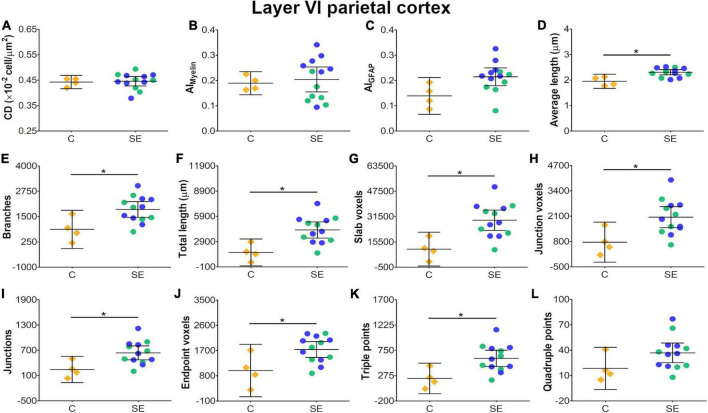
Histological-derived parameters from automated cell counting analyses **(A)**, structure-tensor (ST)-based analyses of myelin **(B)**, and ST **(C)** and skeleton-based analysis **(D–L)** from GFAP-stained sections in layer VI of the parietal cortex. Notations as in Figure 4. SE animals exhibited increases in average length **(D)** and in all skeleton-based parameters **(E–L)** as compared to controls (BH-FDR-corrected *q*-value * < 0.05; two-side permutation *t*-test). AI, anisotropy index; C, control; CD, cell density; SE, status epilepticus.

In layer V of the parietal cortex, the effect of SE was medium in CL and CP (Table 1 and Figures 2E,F). We did not observe any apparent changes in cyto- or myeloarchitecture between SE and control animals (Figures 3A2–D2), but we detected an increase in both the number and the length of the astrocyte processes in SE animals (Figure 3F2) as compared to controls (Figure 3E2) (Figure 3F2). The effect of SE was clear in all extracted skeleton-based parameters (|d| > 0.8; Table 2 and Figures 5E–L) except for average length (Table 2 and Figure 5D), and medium in AI_*Myelin*_ (Table 2 and Figure 5B). Although the effect of SE was medium in CL, CP, and AI_*Myelin*_, CIs were wider in those parameters in this cortical layer, indicating uncertainty about the effect of SE in those parameters. In CD and AI_*GFAP*_, the effect of SE was small (Figures 5A,C). In layer VI of the parietal cortex, the effect of SE was large in FA [|d| = 1.080, 95% CI (−0.59, 2.42)], CP [|d| = 1.250, 95% CI (0.53, 2.12)] and CS [|d| = 1.430, 95% CI (−2.48, −0.01)] (Table 1 and Figures 2A,F,G). Moreover, the effect of SE was medium in AD, but CI revealed extensive variation, and uncertainty about the effect of SE should be considered (Table 1 and Figure 2B). We did not observe any apparent changes in cellularity when comparing control and SE animals (Figures 3A3,B3), but myelinated axons appeared more numerous and aligned in a dorso-ventral orientation (Figures 3C3,D3). There was also an increase in the number and length of astrocyte processes in SE animals (Figures 3E3,F3). In GFAP-stained sections, the ST-based analysis revealed that the effect of SE was large in AI_*GFAP*_ [|d| = 1.350, 95% CI (0.20, 2.70); Table 2 and Figure 6C] and in all skeleton-based parameters (|d| > 0.8; Table 2 and Figures 6D–L). However, the effect of SE was small in CD and AI_*Myelin*_ (Figures 6A,B). Altogether, the effects of SE in AI_*GFAP*_, and skeleton-based parameters, in layers V and VI in the parietal cortex are indicative of changes in the organization and morphology of the astrocyte processes in SE rats.

**FIGURE 7:**
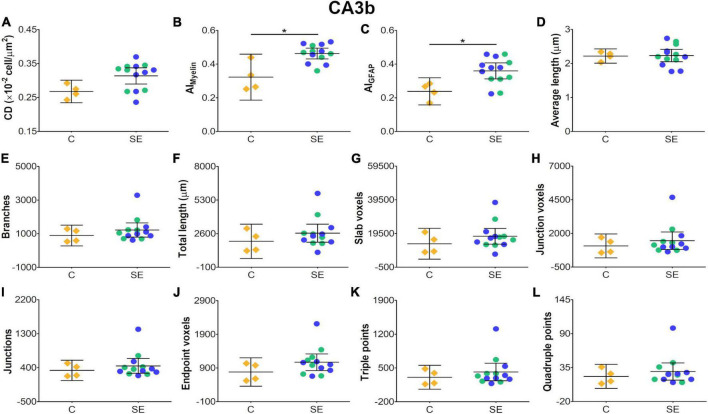
Histological-derived parameters from automated cell counting analyses **(A)**, structure-tensor (ST)-based analyses of myelin **(B)**, and ST **(C)** and skeleton-based analysis **(D–L)** from GFAP-stained sections in the subfield CA3b. Notations as in Figure 4. SE animals revealed increases in AI_*Myelin*_ **(B)** and AI_*GFAP*_ **(C)** parameters as compared to controls (BH-FDR-corrected *q*-value * < 0.05; two-side permutation *t*-test). AI, anisotropy index; C, control; CD, cell density; SE, status epilepticus.

In the CA3b, the effect of SE was large in FA [|d| = 2.090, 95% CI (0.98, 3.22)], AD [|d| = 0.993, 95% CI (−0.26, 2.64)], CL [|d| = 1.150, 95% CI (0.41, 1.92)], CP [|d| = 1.210, 95% CI (0.40, 1.99)] and CS [|d| = −2.560, 95% CI (−3.71, −1.33)] (Table 1 and Figures 2A,B,E–G). It is noteworthy that the effect of SE was large in DTI parameters with narrow CIs, revealing the robustness of the effect evoked by SE. As compared to controls, the animals which had experienced SE exhibited increased cellularity (Figures 3A4,B4), a reorganization of myelinated axons (Figures 3C4,D4), and an increase in the number and length of astrocyte processes (Figures 3E4,F4). The effect of SE was large in CD [|d| = 1.250, 95% CI (0.41, 2.71)], AI_*Myelin*_ [|d| = 2.300, 95% CI (0.45, 4.34)], and AI_*GFAP*_ [|d| = 1.650, 95% CI (0.80, 3.14)] (Table 2 and Figures 7A–C). The effect of SE was medium in the number of branches, total length, slab, and endpoint voxels (Table 2 and Figures 7E–G,J), but the CIs displayed large variation. On the other hand, the effect of SE was small in average length, junction voxels, junctions, triple and quadruple points (Figures 7D,H,I,K,L). Thus, the large effect of SE in AI_*GFAP*_ and AI_*Myelin*_ suggested reorganization of both myelinated axons and astrocyte processes in the CA3b after SE. In DTI parameters such as RD and MD, we did not find the effect of SE in any of the areas analyzed in this study (Figures 2C,D).

### Relationship between histological and diffusion tensor imaging parameters

**FIGURE 8:**
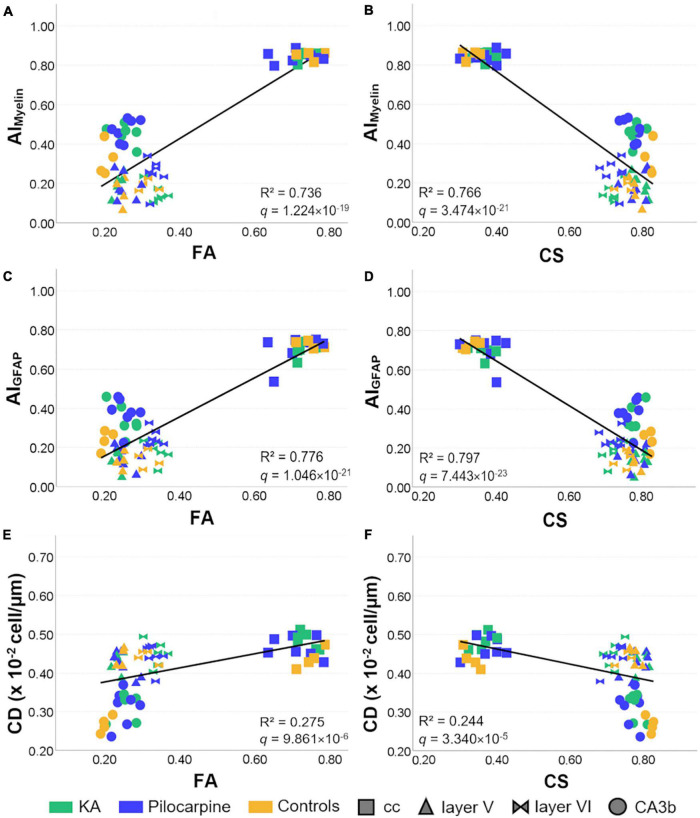
Representative relationships between DTI and histological parameters in all selected brain regions. The line represents the regression fit between histological and DTI parameters. Controls and status epilepticus animals are represented by colors, while brain regions by shapes. FA and CS showed large effects in AI_*Myelin*_ and AI_*GFAP*_ **(A–D)**, while medium in CD **(E,F)** when analyzing the relationships between DTI and histological parameters individually. *R*^2^ and BH-FDR corrected *q*-values for the univariate Pearson’s correlation between DTI and histological parameters are shown in each graph. FA, fractional anisotropy; AI, anisotropy index; CA, cornus ammonis; cc, corpus callosum; CD, cell density; CS, spherical anisotropy.

When using the multivariate DTI model, we found that AI_*Myelin*_ was extremely well explained by DTI parameters [*R*^2^ = 0.822, R2 adj = 0.807, 95% CI (0.72, 0.86); Table 3], where FA, RD, and CS showed large effects [*R*^2^ > 0.60, R > 0.80; Table 4 and Figures 8A,B] according to the univariate DTI model. Similarly to AI_*Myelin*_, DTI parameters explained AI_*GFAP*_ well [*R*^2^ = 0.855, R^2^ adj = 0.843, 95% CI (0.77, 0.88); Table 3], whereas FA, RD, and CS also exhibited large effects [*R*^2^ > 0.65, R > 0.80; Table 4 and Figures 8C,D]. In CD, over 50% of the variation was explained by the multivariate DTI model [*R*^2^ = 0.557, R^2^ adj = 0.521, 95% CI (0.35, 0.64); Table 3], whereas the univariate DTI model indicated that FA, RD, and CS parameters also showed medium effects [*R*^2^ > 0.20, R > 0.45; Table 4 and Figures 8E,F). Moreover, all skeleton-based and DTI parameters correlated, except for average length; in these cases, DTI explained over 20% of the variation in these histological parameters (Table 3). In more detail, DTI parameters explained over 60% of the variation in endpoint voxels [*R*^2^ = 0.659, R^2^ adj = 0.632, 95% CI (0.48, 0.73); Table 3], and over 50% of the variation in branches [*R*^2^ = 0.507, R^2^ adj = 0.467, 95% CI (0.29, 0.60); Table 3], whereas FA, RD, and CS exhibited medium effects (*R*^2^ > 0.40, R > 0.60; Table 4). Altogether, these findings indicate that the multivariate DTI model explained the histological parameters better than the univariate DTI models.

**TABLE 3:** Multiple linear regression analyses between DTI and histological parameters and leave-one-animal out cross-validation.

	*R*^2^ (95% CI) *q*	R^2^ adj.	F	R *Q*^2^
AI_*Myelin*_	0.822 (0.72, 0.86)*** 2.349 × 10^–20^	0.807	57.076	0.876 0.766
AI_*GFAP*_	0.855 (0.77, 0.88)*** 8.038 × 10^–23^	0.843	73.086	0.909 0.825
CD	0.557 (0.35, 0.64)*** 1.176 × 10^–8^	0.521	15.585	0.489 0.050
Average length	0.104 (0.00, 0.20) 0.331	0.032	1.439	−0.014 −0.161
Branches	0.507 (0.29, 0.60)*** 2.238 × 10^–7^	0.467	12.758	0.207 −15.160
Branch length	0.467 (0.24, 0.57)*** 1.856 × 10^–6^	0.424	10.874	0.209 −15.040
Slab voxels	0.453 (0.22, 0.56)*** 3.452 × 10^–6^	0.409	10.268	0.206 −14.984
Junction voxels	0.326 (0.10, 0.44)** 0.001	0.272	6.008	0.114 −18.482
Junctions	0.317 (0.09, 0.43)** 0.001	0.262	5.750	0.113 −17.735
Endpoint voxels	0.659 (0.48, 0.73)*** 5.871 × 10^–12^	0.632	23.977	0.296 −11.236
Triple points	0.322 (0.10, 0.44)** 0.001	0.268	5.895	0.116 −17.569
Quadruple points	0.251 (0.04, 0.37)* 0.014	0.191	4.158	0.062 −19.835

Multivariate DTI regression model (R^2^) strongly explained AI_*Myelin*_ and AI_*GFAP*_ by DTI, while DTI explained 50 and 20% of the variation in CD and all skeleton-based parameters, respectively. Cross-validation of the model by leaving-one-animal out (R) indicated that DTI accurately predicted AI_*Myelin*_ and AI_*GFAP*_, while moderately CD. BH-FDR-corrected *q*-values for multiple linear regression tests between DTI and histological parameters are denoted with asterisks (*q < 0.05; **q < 0.01; ***q < 0.001; multiple linear regression model). AI, anisotropy index; CD, cell density; CI, confidence intervals.

**TABLE 4:** Pearson’s correlations analyses between DTI and histological parameters.

	FA	RD	MD	CP	CS

	R (95% CI) *R*^2^ *q*	R (95% CI) *R*^2^ *q*	R (95% CI) *R*^2^ *q*	R (95% CI) *R*^2^ *q*	R (95% CI) *R*^2^ *q*
AI_*Myelin*_	0.858 (0.77, 0.91)*** 0.736 1.224 × 10^–19^	−0.805 (−0.87, −0.71)*** 0.648 1.300 × 10^–15^	0.484 (0.30, 0.63)*** 0.234 5.111 × 10^–5^	0.584 (0.40, 0.73)*** 0.341 5.418 × 10^–7^	−0.875 (−0.92, −0.81)*** 0.766 3.474 × 10^–21^
AI_*GFAP*_	0.881 (0.81, 0.93)*** 0.776 1.046 × 10^–21^	−0.817 (−0.88, −0.72)*** 0.667 2.235 × 10^–16^	0.535 (0.36, 0.68)*** 0.286 6.576 × 10^–6^	0.563 (0.40, 0.71)*** 0.317 1.672 × 10^–6^	−0.893 (−0.94, −0.82)*** 0.797 7.443 × 10^–23^
CD	0.524 (0.41, 0.62)*** 0.275 9.861 × 10^–6^	−0.575 (−0.68, −0.46)*** 0.331 8.502 × 10^–7^	−0.113 (−0.35, 0.09) 0.013 0.359	0.161 (−0.05, 0.34) 0.026 0.196	−0.494 (−0.61, −0.36)*** 0.244 3.340 × 10^–5^
Average length	−0.278 (−0.46, 0.09)* 0.077 0.026	0.251 (0.06, 0.43)* 0.063 0.043	−0.151 (−0.37, 0.07) 0.023 0.223	−0.238 (−0.46, 0.02) 0.057 0.055	0.285 (0.10, 0.48)* 0.081 0.023
Branches	0.677 (0.50, 0.80)*** 0.458 1.387 × 10^–9^	−0.647 (−0.76, −0.50)*** 0.419 1.231 × 10^–8^	0.362 (0.14, 0.56)** 0.131 0.004	0.331 (0.09, 0.56)** 0.110 0.008	−0.673 (−0.79, −0.51)*** 0.453 1.807 × 10^–9^
Branch length	0.645 (0.46, 0.78)*** 0.416 1.341 × 10^–8^	−0.616 (−0.75, −0.42)*** 0.379 7.766 × 10^–8^	0.345 (0.12, 0.53)** 0.119 0.006	0.301 (0.01, 0.56)* 0.091 0.017	−0.638 (−0.76, −0.48)*** 0.407 2.013 × 10^–8^
Slab voxels	0.632 (0.47, 0.78)*** 0.399 2.885 × 10^–8^	−0.603 (−0.73, −0.45)*** 0.364 1.705 × 10^–7^	0.342 (0.11, 0.54)** 0.117 0.006	0.292 (0.03, 0.55)* 0.085 0.020	−0.625 (−0.76, −0.46)*** 0.391 4.463 × 10^–8^
Junction voxels	0.540 (0.36, 0.68)*** 0.292 5.364 × 10^–6^	−0.513 (−0.67, −0.33)*** 0.263 1.528 × 10^–5^	0.297 (0.08, 0.49)* 0.088 0.018	0.259 (−0.001, 0.50)* 0.067 0.038	−0.535 (−0.69, −0.33)*** 0.286 6.576 × 10^–6^
Junctions	0.529 (0.33, 0.70)*** 0.280 8.246 × 10^–6^	−0.500 (−0.67, −0.31)*** 0.250 2.683 × 10^–5^	0.298 (0.06, 0.51)* 0.089 0.018	0.255 (0.003, 0.50)* 0.065 0.040	−0.523 (−0.68, −0.32)*** 0.273 9.896 × 10^–6^
Endpoint voxels	0.774 (0.66, 0.86)*** 0.599 8.530 × 10^–14^	−0.745 (−0.83, −0.65)*** 0.555 2.192 × 10^–12^	0.400 (0.19, 0.58)** 0.160 0.001	0.383 (0.16, 0.60)** 0.147 0.002	−0.771 (−0.85, −0.67)*** 0.594 1.042 × 10^–13^
Triple points	0.533 (0.31, 0.71)*** 0.284 7.047 × 10^–6^	−0.502 (−0.66, −0.30)*** 0.252 2.475 × 10^–5^	0.303 (0.10, 0.50)* 0.092 0.016	0.261 (−0.003, 0.52)* 0.068 0.037	−0.527 (−0.68, −0.34)*** 0.278 8.739 × 10^–6^
Quadruple points	0.468 (0.25, 0.64)*** 0.219 9.796 × 10^–5^	−0.456 (−0.63, −0.25)*** 0.208 1.502 × 10^–4^	0.228 (0.05, 0.42) 0.052 0.065	0.180 (−0.07, 0.42) 0.032 0.149	−0.461 (−0.65, −0.23)*** 0.212 1.254 × 10^–4^

Univariate DTI analysis by Pearson’s correlation highlighted that FA, RD, and CS showed strong correlations with histological parameters. BH-FDR-corrected q-values for Pearson’s correlations between DTI and histological parameters are denoted with asterisks (*q < 0.05; **q < 0.01; ***q < 0.001; Pearson’s correlation). AI, anisotropy index; CD, cell density; CI, confidence intervals; CP, planar anisotropy; CS, spherical anisotropy; FA, fractional anisotropy; MD, mean diffusivity; RD, radial diffusivity.

The CV analysis revealed the strong predictive accuracy of DTI parameters for assessing AI_*Myelin*_ (R = 0.876; *Q*^2^ = 0.766) and AI_*GFAP*_ (R = 0.909; *Q*^2^ = 0.825), and moderate for CD (R = 0.489; *Q*^2^ = 0.050) when applying the approach of leaving-one-animal out (Table 3). We also found that the model did not have a predictive value for skeleton-based parameters (R > 0.05; *Q*^2^ > −11.00; Table 3). Moreover, a leave-one-brain region out CV analysis indicated that the inclusion of subfield CA3b was necessary if one wished to obtain strong predictive relationships between DTI and histological parameters (Table 5).

**TABLE 5:** Leave-one-brain region out cross-validation of multiple linear regression analyses between DTI and histological parameters.

	All brain regions	cc	layer V	layer VI	CA3b
	
	R	R	R	R	R
AI_*Myelin*_	0.206	0.289	0.049	0.353	0.557
AI_*GFAP*_	0.226	0.030	0.097	−0.045	0.206
CD	0.157	−0.088	−0.217	−0.014	0.300
Average length	−0.002	0.045	−0.223	−0.019	−0.050
Branches	0.154	−0.059	0.023	0.125	−0.071
Branch length	0.165	0.358	0.288	0.020	0.168
Slab voxels	0.165	0.055	0.032	−0.034	−0.456
Junction voxels	0.108	0.185	0.236	0.353	0.384
Junctions	0.107	−0.016	−0.063	0.132	0.441
Endpoint voxels	0.190	−0.016	0.039	0.020	0.228
Triple points	0.109	0.086	0.072	0.007	0.234
Quadruple points	0.070	−0.059	0.001	−0.022	0.242

Cross-validation by leaving-one-brain region out (R) revealed that the inclusion of the subfield CA3b was necessary for predicting histology based on DTI. AI, anisotropy index; CA, cornus ammonis; cc, corpus callosum; CD, cell density.

## Discussion

In this study, we investigated the association of *in vivo* DTI parameters to one and/or several changes in histological parameters after SE-induced brain damage and examined the prediction of underlying tissue changes based on DTI. When comparing SE animals to controls, changes in *in vivo* DTI parameters in the corpus callosum might be associated with changes in cellularity and morphology of astrocyte processes. In the parietal cortex, these alterations might be related to changes in the morphology of astrocyte processes in layer V, as well as in the morphology and organization of astrocyte processes in layer VI. In CA3b, changes in *in vivo* DTI parameters might associate with morphometric changes in all the cellular components analyzed in this study. More importantly, we found that *in vivo* DTI parameters were highly predictive of AI_*Myelin*_ and AI_*GFAP*_, moderately predictive of CD, but not predictive for skeleton-based parameters. Furthermore, we found that it was necessary to include the CA3b brain region when modeling histopathology based on DTI. In this regard, this study indicates that a multivariate DTI model approach provides a better explanation of histological parameters than any single univariate model. Altogether, our results represent a step forward in the interpretation of DTI parameters, showing how they potentially reflect the underlying changes in the rat brain’s tissue microstructure after SE.

Previous studies have reported changes in DTI parameters in the corpus callosum after SE, e.g., either an increase (Sierra et al., 2011) or a decrease in FA (Luna-Munguia et al., 2021), as well as a decrease in AD (van Eijsden et al., 2011; Luna-Munguia et al., 2021), an increase in RD (Luna-Munguia et al., 2021) and a decrease in MD (van Eijsden et al., 2011). Here, we found that the effects of SE in DTI parameters revealed a large effect in CS, and medium in FA and AD with wide CIs that indicate uncertainty about the effects of SE, but altogether the changes in these DTI parameters might associate with an increase in cellularity and morphological changes of astrocyte processes in the corpus callosum. However, no apparent changes were observed in myelinated axons. van Eijsden et al. (2011) examined the changes in the corpus callosum at 4 and 8 weeks after SE using the pilocarpine model. These authors found a transient decrease in MD at 4 weeks and a persistent decrease in AD at 4 and 8 weeks post-SE, related to a decrease and partial recovery in myelin intensity, respectively. No apparent changes in myelinated axons found in the previous and present study at the chronic phase might indicate axonal alterations in the early stages after SE with recovery over time. However, in this study, Nissl and GFAP stainings revealed evidence of still active and ongoing inflammatory processes at 79 days. These results may be indicative of ongoing inflammation without axonal alterations in the corpus callosum at this timepoint after SE. Thus, these results also indicate that not only myelinated axons can modify the microstructural environment, but those other cellular contributors, such as inflammatory cells, can also influence DTI parameters, in agreement with previous reports in other white matter regions (Sierra et al., 2011; Luna-Munguia et al., 2021).

Reactive glial cells change their morphology and increase the number and length of their processes. Budde et al. (2011) performed a quantitative Fourier transform-based histological analysis after an experimental traumatic brain injury and reported that increased FA in the cortex was associated with changes in the structural organization of reactive astrocytes. In the present study, we also associated changes in *in vivo* DTI parameters with changes in astrocyte processes at 79 days post-SE. In layer V of the parietal cortex, we hypothesized that the changes in the morphology of astrocyte processes might relate to the medium effects of SE in DTI parameters such as CL and CP, although the CIs revealed uncertainty about these effects. In layer VI, the large effect of SE in FA, CP, and CS might associate with changes in both the organization and morphology of astrocyte processes, but uncertainty about the effects of SE in FA and CS should be considered since wide CIs were revealed. It is noteworthy that although we did not detect any effect of SE in the organization of myelinated axons in layer VI, our findings revealed a greater number, which was more aligned in the dorso-ventral orientation in SE animals. Therefore, our findings indicate that the reorganization of astrocytes and myelinated axons in the cortex can be reflected in DTI parameters after SE.

Several studies have described increased FA in the hippocampus and dentate gyrus associated with axonal plasticity in rats (Kuo et al., 2008; Laitinen et al., 2010; Parekh et al., 2010; Sierra et al., 2011, 2015; Salo et al., 2017). It has been reported that increases in FA and CP and a decrease in CS in both the CA3b and CA3c subfields were related to changes in the reorganization of myelinated axons and astrocyte processes (Salo et al., 2017), and a decreased FA has been linked to neurodegeneration and microglial scarring of the pyramidal cell layer in the CA1 (Sierra et al., 2015; Janz et al., 2017). Here, we found that the effect of SE in FA, AD, CL, CP, and CS, might be associated with an increase in CD, and a reorganization of myelinated axons and astrocytes in CA3b. It is important to highlight that AD and CS showed wider CI and should be considered the uncertainty of the effects of SE in this regard. Thus, previous and current findings suggest that changes in both axons and astrocytes can be detected in the hippocampus by DTI in rats after SE.

Our findings highlight that DTI parameters were substantially associated with anisotropy of myelinated axons and astrocyte processes in white and gray matter areas. Moreover, a multivariate DTI model provided a better explanation of the histological parameters as compared to the univariate models. Our CV of the regression model confirmed that DTI parameters were very predictive of these histological changes in white and gray matter areas at 79 days post-SE. Furthermore, DTI moderately explained CD although it was less useful in explaining skeleton-based parameters. Regarding the selected brain regions, we found that the predictive models, which did not include data from CA3b, were much less accurate than those including this brain region. This indicates that one needs to have sufficient heterogeneity of quantitative parameters if the intent is to build predictive models of histological parameters. Modeling approaches focusing exclusively on a single brain region might not be successful.

There are a few limitations in our study that should be considered. First, based on the CV of DTI and histology, future studies should increase the number of animals and brain regions. As we demonstrated that including CA3b achieved a better prediction of histological parameters based on DTI when compared to other brain regions. In this regard, the presence of wide confidence intervals is also an indication that increasing the sample size would be one way to improve the estimations about the impact of SE when assessing changes in both DTI and histology. Second, as only a limited number of diffusion directions were measured with relatively low-*b*-value, we used a tensor model of diffusion and this may underestimate the interpretation of complex alterations in tissue microstructure (Jeurissen et al., 2013). Therefore, more advanced dMRI methods and post-processing tools *in vivo* might improve the detection of tissue changes under pathological conditions (Tournier et al., 2004, 2007; Zhang et al., 2012; Jeurissen et al., 2014; Topgaard, 2017; de Almeida Martins et al., 2021). Third, it is also important to highlight the partial volume effect when comparing DTI and histology parameters extracted by ROI analysis. The different resolutions in DTI (110 × 110 × 500 μm^3^) and histology (0.114 × 0.114 μm^2^) might influence the delineation of ROIs and the outcomes when comparing both methodologies. Also, larger ROIs outlined in DTI maps might exhibit more microstructural heterogeneity than the representative ROIs in photomicrographs, affecting the effect sizes of SE and the relationship between DTI and histological parameters. Moreover, the effects of chemical fixation and staining procedures can affect the tissue microstructure, and thus, the comparisons between *in vivo* DTI and *ex vivo* histological parameters (Sun et al., 2003; Howard et al., 2019). Fourth, we compared a 2D histological assessment against 3D *in vivo* DTI data. Thus, 3D quantitative histological analyses can provide a more reliable comparison with DTI (Khan et al., 2015; Morawski et al., 2018; Schilling et al., 2018; Salo et al., 2021). Furthermore, automated cell counting- and quantitative skeleton-based analyses are useful to extract CD and morphology information from the tissue, respectively; however, these algorithms might require further development to increase their accuracy. Finally, the focus of our study was on the relationship between histopathology and DTI in SE-induced brain damage, a similar approach can be utilized when studying other brain diseases and disorders by combining other MRI methods, several cell markers, or even different methodologies, such as electrophysiology or behavioral data. For example, to fully characterize resting and activated astrocytes, additional specific markers should be considered (Jurga et al., 2021). Also, future studies including other glial cell markers could associate the full inflammatory response post-SE to DTI parameters. These additions can improve the robustness of our predictive model and the CV approach after SE, or when implementing this type of analysis in other disease models or human studies.

In conclusion, our findings suggest that *in vivo* DTI can be predictive of quantitative tissue microstructure parameters estimated from cellularity, organization of myelinated axons, and morphology of astrocyte processes in white and gray matter areas after SE-induced brain damage. This study offers new insights to help in the interpretation of *in vivo* DTI as it incorporated an advanced quantitative histological assessment and CV of DTI and histology. Future studies combining imaging and histology with advanced analytical tools are needed to improve our understanding of imaging outcomes in terms of tissue microstructural mechanisms during pathological conditions and to open new perspectives in the diagnosis and prognosis of brain diseases.

## Data availability statement

The raw data supporting the conclusions of this article will be made available by the authors, without undue reservation.

## Ethics statement

All animal procedures were approved by the Animal Ethics Committee of the Provincial Government of Southern Finland and performed in accordance with the guidelines set by the European Community Council Directives 2010/63/EEC.

## Author contributions

ISMM, JT, and AS designed the experiment. ISMM, RAS, and JT analyzed the data. ISMM, RAS, JT, OG, and AS wrote the manuscript. All authors contributed to the article and approved the submitted version.

## Footnotes

1
http://www.fmrib.ox.ac.uk/fsl/

2
https://github.com/aAbdz/cell_counter

3
https://imagej.net/AnalyzeSkeleton

4
http://aedes.uef.fi/

5
https://github.com/jussitohka/mrihistology

## Supplementary material

Supplementary Figure 1 Representative images of the automated cell counting-based approach in Nissl-stained sections in the corpus callosum, layers V and VI of the parietal cortex, and CA3b of a control animal. Counted cells are highlighted with red dots by the automated cell counting-based approach. Scale bar: 50 μm. cc, corpus callosum; CA, cornus ammonis.

Supplementary Figure 2 Representative images of the skeleton-based approach in GFAP-stained sections in the corpus callosum, layer V and VI of the parietal cortex, and CA3b of a control animal. Astrocyte morphology is delineated in black by the skeleton-based approach. Scale bar: 50 μm. cc, corpus callosum; CA, cornus ammonis.

## Abbreviations

AD: axial diffusivity
AI: anisotropy index
CD: cell density
CI: confidence intervals
CL: linear anisotropy
CP: planar anisotropy
CS: spherical anisotropy
CV: cross-validation
dMRI: diffusion magnetic resonance imaging
DTI: diffusion tensor imaging
FA: fractional anisotropy
GFAP: glial fibrillary acidic protein
MD: mean diffusivity
MRI: magnetic resonance imaging
RD: radial diffusivity
ROI: region-of-interest
SE: status epilepticus
ST: structure tensor.
