# Supplementary figures and images for: Histopathological modeling of status epilepticus-induced brain damage based on in vivo diffusion tensor imaging in rats
Source: Front Neurosci. 2022 Jul 29;16:944432. doi: 10.3389/fnins.2022.944432 (PMC9372371; doi:10.3389/fnins.2022.944432)

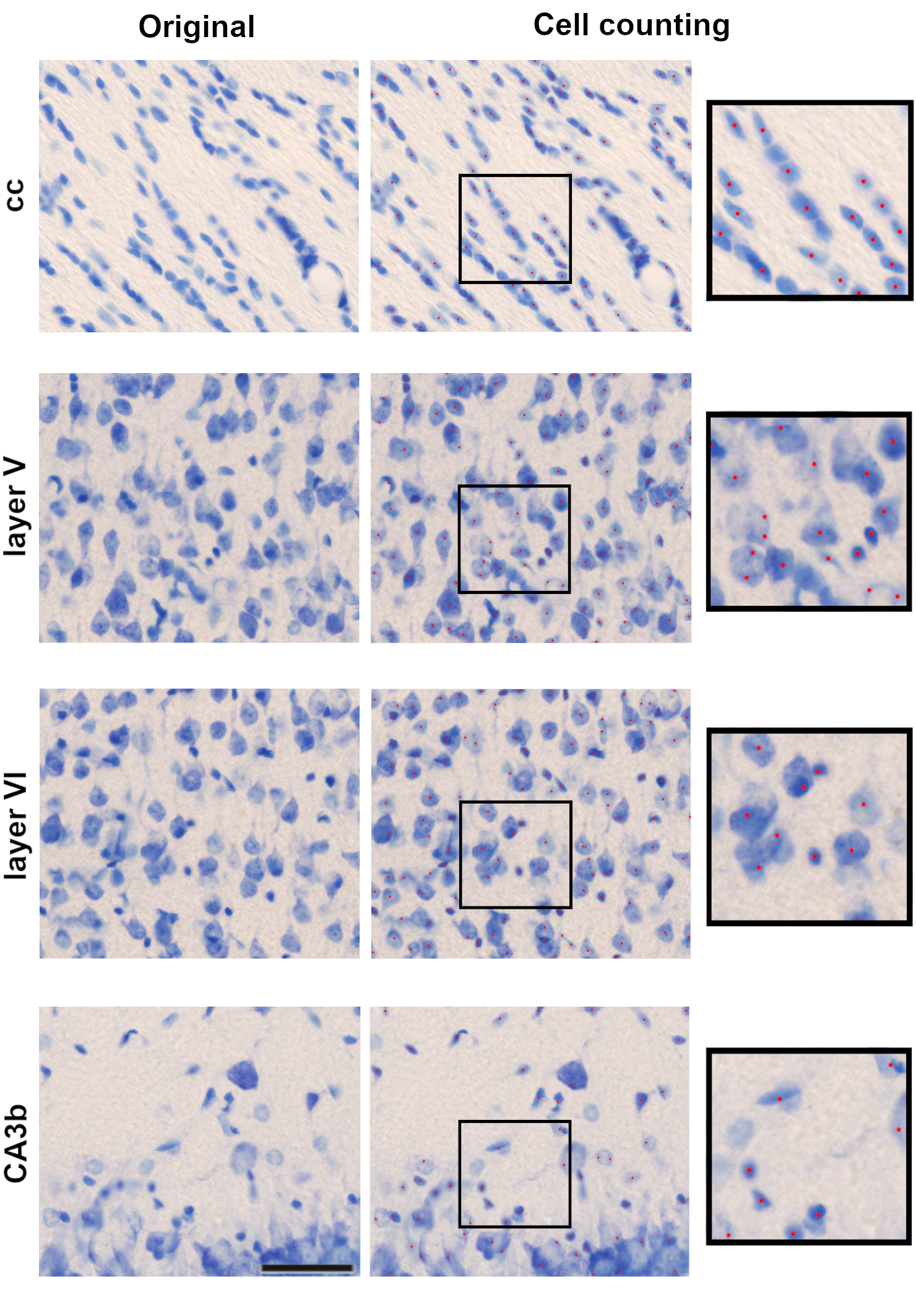

Supplement: Supplementary Figure 1 — Representative images of the automated cell counting-based approach in Nissl-stained sections in the corpus callosum, layers V and VI of the parietal cortex, and CA3b of a control animal. Counted cells are highlighted with red dots by the automated cell counting-based approach. Scale bar: 50 μm. cc, corpus callosum; CA, cornus ammonis. [file Image_1.TIF]

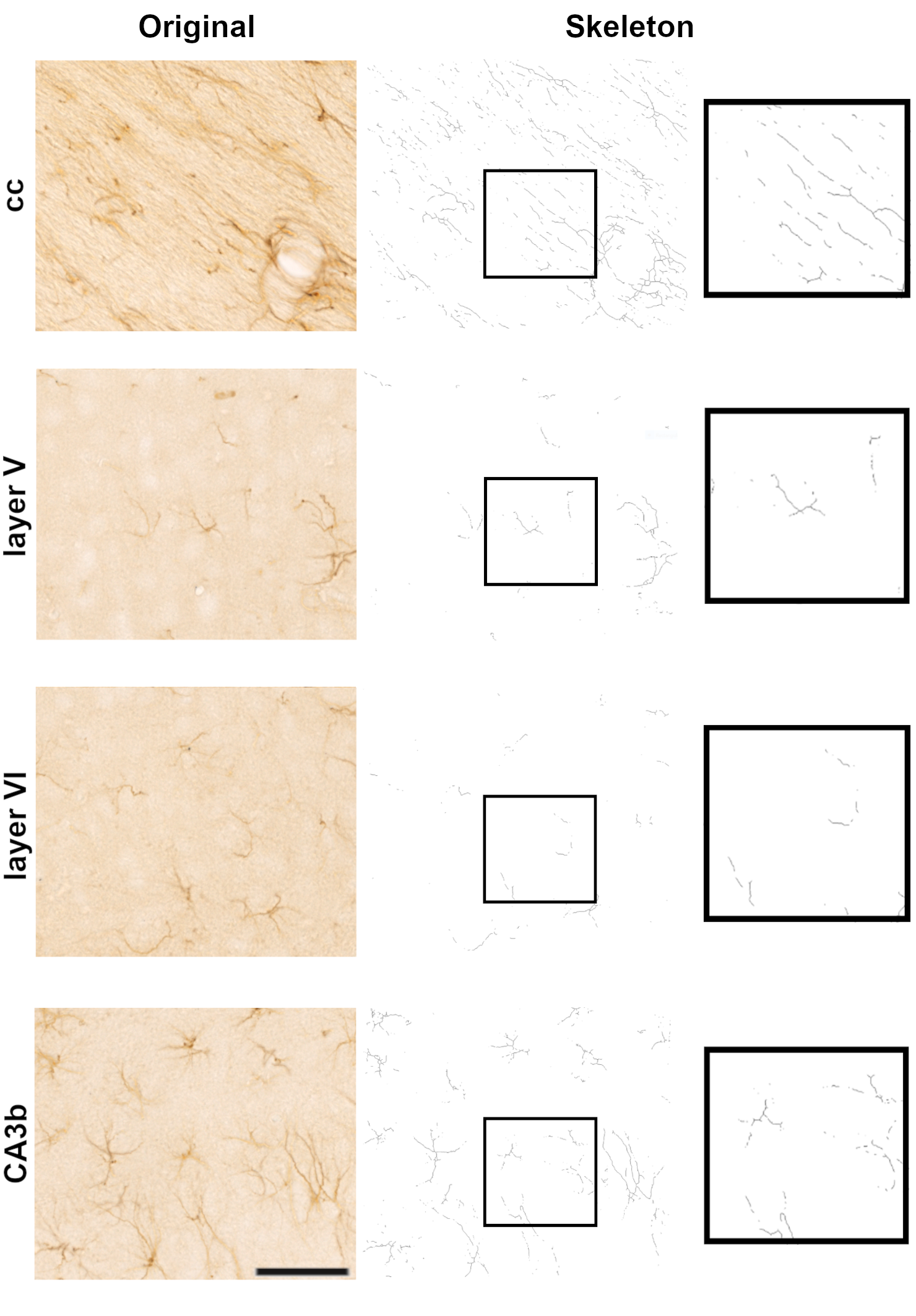

Supplement: Supplementary Figure 2 — Representative images of the skeleton-based approach in GFAP-stained sections in the corpus callosum, layer V and VI of the parietal cortex, and CA3b of a control animal. Astrocyte morphology is delineated in black by the skeleton-based approach. Scale bar: 50 μm. cc, corpus callosum; CA, cornus ammonis. [file Image_2.TIF]
